# The exon junction complex is required for definition and excision of neighboring introns in *Drosophila*

**DOI:** 10.1101/gad.245738.114

**Published:** 2014-08-15

**Authors:** Rippei Hayashi, Dominik Handler, David Ish-Horowicz, Julius Brennecke

**Affiliations:** 1 Institute of Molecular Biotechnology of the Austrian Academy of Sciences (IMBA), 1030 Vienna, Austria;; 2 MRC Laboratory for Molecular Cell Biology, University College London, London WC1E 6BT, United Kingdom

**Keywords:** Piwi–piRNA pathway, SR proteins Acinus and RnpS1, exon junction complex, intron definition, splicing, transposon silencing

## Abstract

Splicing of pre-mRNAs results in the deposition of the exon junction complex (EJC) upstream of exon–exon boundaries. The EJC plays crucial post-splicing roles in export, translation, localization, and nonsense-mediated decay of mRNAs. It also aids faithful splicing of pre-mRNAs containing large introns, albeit via an unknown mechanism. Here, we show that the core EJC plus the accessory factors RnpS1 and Acinus aid in definition and efficient splicing of neighboring introns. This requires prior deposition of the EJC in close proximity to either an upstream or downstream splicing event. If present in isolation, EJC-dependent introns are splicing-defective also in wild-type cells. Interestingly, the most affected intron belongs to the *piwi* locus, which explains the reported transposon desilencing in EJC-depleted *Drosophila* ovaries. Based on a transcriptome-wide analysis, we propose that the dependency of splicing on the EJC is exploited as a means to control the temporal order of splicing events.

## Abstract (precis)

The exon junction complex (EJC) plays a central role in controlling RNA fate and aids faithful splicing of pre-mRNAs containing large introns via an unknown mechanism. Brennecke and colleagues show that the core EJC plus the accessory factors RnpS1 and Acinus aid in the definition and efficient splicing of neighboring introns. Interestingly, the most highly affected intron belongs to the *piwi* locus, which explains the reported transposon desilencing in EJC-depleted *Drosophila* ovaries. Based on transcriptome-wide analysis, the authors propose that the dependency of splicing on the EJC is exploited to control the temporal order of splicing events.

Expression of protein-coding genes involves a series of interlinked and interdependent molecular events, including nuclear steps such as pre-mRNA transcription, intron removal via splicing, 5′ and 3′ modifications, and export, followed by cytoplasmic events such as mRNA translation and degradation. A central factor connecting nuclear pre-mRNA maturation to mRNA fate is the exon junction complex (EJC), a multisubunit protein complex that is deposited ∼24 nucleotides (nt) upstream of individual exon–exon boundaries after splicing (Le Hir et al. 2000a).

Assembly of the EJC on an RNA depends on tight RNA binding of a trimeric nuclear complex of the sequence-independent DEAD-box RNA clamp eIF4AIII and a heterodimer of Mago and Y14/Tsunagi (Tsu) (Shibuya et al. 2004; Gehring et al. 2009). Upon nuclear mRNA export to the cytoplasm, these three factors recruit a fourth EJC core component, Barentsz (Btz)/MLN51 (Degot et al. 2004).

The EJC is a binding platform for several peripheral factors, which provide mechanistic links to various processes in mRNA fate control (Tange et al. 2004; Bono and Gehring 2011). Among these are the nuclear mRNA export factors UAP56, REF/Aly, and TAP/NFX1:p15 (Kataoka et al. 2000; Luo et al. 2001); the nonsense-mediated mRNA decay factor Upf3 (Gehring et al. 2003); and the ASAP complex (Acinus [Acn], RnpS1, and SAP18), whose subunits have been individually implicated in mRNA quality control, pre-mRNA splicing, and transcriptional regulation (Zhang et al. 1997; Mayeda et al. 1999; Schwerk et al. 2003; Sakashita et al. 2004).

Most molecular processes that depend on the EJC are post-splicing events. However, the EJC core also interacts with proteins implicated in splicing (e.g., the splicing coactivators/alternative splicing factors SRm160 and Pinin) (Le Hir et al. 2000b; Li et al. 2003), suggesting that the EJC plays a direct role in splicing itself. Indeed, the nuclear EJC core factors eIF4AIII, Mago, and Tsu, together with RnpS1, are required for the faithful splicing of several *Drosophila* pre-mRNAs harboring large introns, often encoded at heterochromatic gene loci (Ashton-Beaucage et al. 2010; Roignant and Treisman 2010). Loss of the EJC results in frequent exon skipping and reduced mRNA levels, the most prominent example being the ERK-encoding *rolled* transcript.

It is unclear how the EJC facilitates splicing mechanistically—in particular, whether the EJC aids splicing of neighboring introns after it has been deposited at flanking spliced junctions or whether individual EJC subunits directly facilitate splicing independently of their assembly into an EJC.

Here, we demonstrate that the nuclear EJC core factors Mago, Tsu, and eIF4AIII and the peripheral EJC components RnpS1 and Acn are essential for the accurate transcript splicing of *piwi*, which encodes an essential component of the ovarian defense pathway for transposable element (TE) silencing in *Drosophila*. We show that the nuclear EJC is required for splicing of *intron4* of *piwi* and that expression of *piwi* transcripts lacking this intron is EJC-independent. In the absence of the EJC recruited to a flanking exon–exon junction, *intron4* is inefficiently spliced, even in wild-type cells. A transcriptome-wide analysis reveals that several other mRNAs also exhibit an intron retention phenotype upon EJC depletion, indicating that the EJC facilitates the removal of adjacent/nearby introns with suboptimal splicing characteristics.

## Results

### The nuclear EJC is required for transposon silencing in ovaries

TE silencing in *Drosophila* ovarian tissues in both the germline and associated soma is mediated by the piRNA pathway, a small RNA silencing system. Three Argonaute proteins of the PIWI clade (nuclear Piwi and cytoplasmic Aubergine and AGO3) are at its core. Each is bound to a population of 22- to 30-nt single-stranded piRNAs that act as sequence-specific guides to identify complementary targets for their transcriptional and post-transcriptional silencing (Senti and Brennecke 2010; Siomi et al. 2011).

Recent reverse genetic screens have begun to identify the genetic framework of the *Drosophila* piRNA pathway (Czech et al. 2013; Handler et al. 2013; Muerdter et al. 2013). Besides factors involved in piRNA biogenesis or piRNA-mediated silencing, several unexpected players were identified. Among these are components of the EJC, whose depletion results in desilencing of piRNA pathway repressed TEs.

**Figure 1.**
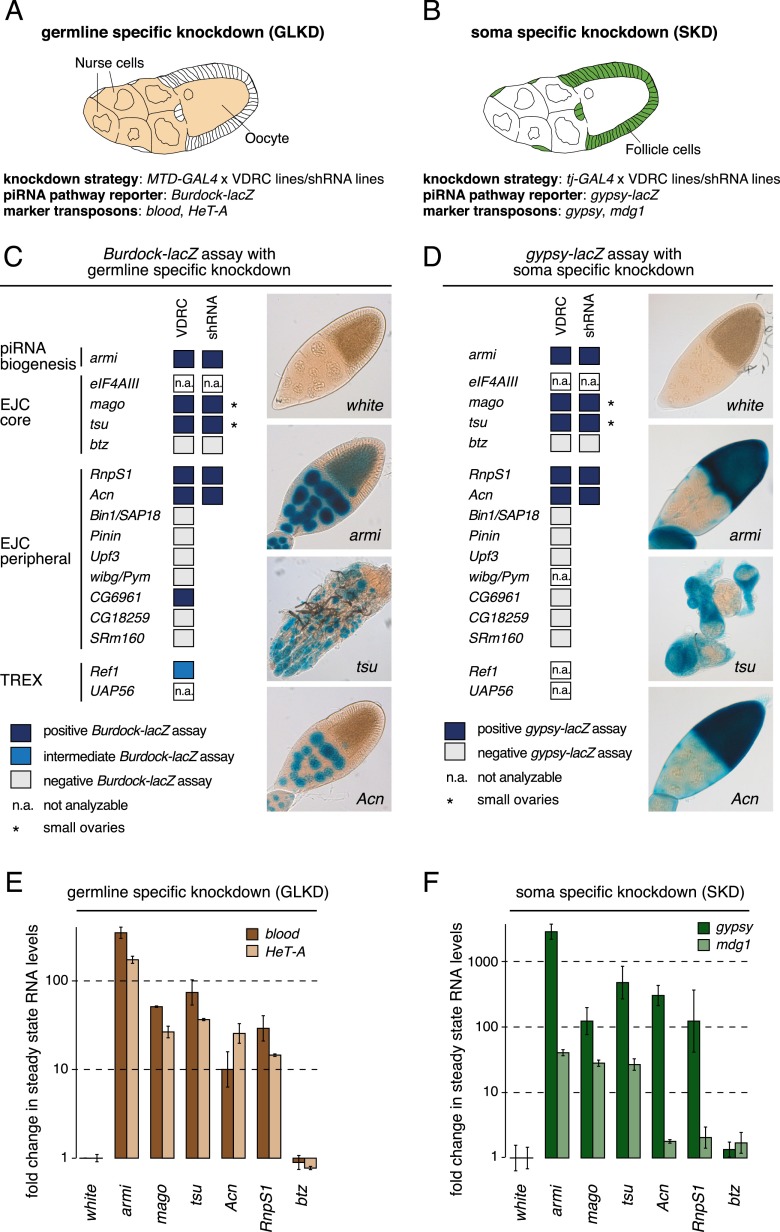
The EJC is required for transposon silencing in ovarian soma and the germline. (*A*,*B*) Cartoons of *Drosophila* egg chambers to illustrate the tissue-specific gene depletions in germline (*A*) or somatic (*B*) cells. The affected cell types (colored), knockdown strategies, TE reporters, and marker TEs expressed in the respective tissues are indicated. (*C*,*D*) The results of the TE reporter assays upon germline (*C*) or soma (*D*) knockdown of EJC factors. Defective TE silencing is reflected by *lacZ* expression (blue). The depletion of EJC core factors *mago* and *tsu*, but not *btz*, as well as the depletion of peripheral factors *RnpS1* or *Acn* consistently leads to *lacZ* expression in both soma and the germline. Stained egg chambers of representative phenotypes are shown: *white* for no staining, *tsu* for strong staining but abnormal morphology, and *Acn* for strong staining and no morphological defects. Knockdown of piRNA biogenesis factor *armi* served as control. (*E*,*F*) Fold changes in steady-state RNA levels of endogenous TEs in ovaries upon depletion of the indicated genes in the germline (*E*) or soma (*F*). RNA levels are normalized to *rp49* levels, and values indicate averages of three biological replicates relative to control knockdowns; error bars indicate standard deviation. The depletion of EJC core factors (*mago* and *tsu*) and peripheral factors (*RnpS1* and *Acn*) causes strong TE derepression almost comparable with *armi* depletions.

We retested 15 annotated core and peripheral EJC factors for a role in ovarian TE silencing. Using tissue-specific RNAi (Dietzl et al. 2007; Ni et al. 2011), we depleted individual factors in either germline tissue (which comprises accessory nurse cells interconnected with each other and with the oocyte) or the overlying somatic follicular epithelium (Fig. 1A,B; Materials and Methods). piRNA pathway integrity was monitored with *lacZ* reporter transgenes that are repressed by the pathway in wild-type cells (Fig. 1C,D) and via quantitative RT–PCR (qRT–PCR) to measure steady-state RNA levels of marker TEs (Fig. 1E,F; Materials and Methods). For both assays, depletion of the essential piRNA biogenesis factor Armitage (Armi) served as positive control.

Only the EJC core factors Mago and Tsu and the peripheral factors RnpS1 and Acn give strong, consistent derepression of the reporters and endogenous TEs in both the ovarian germline (*blood* and *HeT-A*) (Fig. 1C,E) and somatic cells (*gypsy* and *mdg1*) (Fig. 1D,F). Depletion of the core EJC factor eIF4AIII prevents ovarian development entirely and could not be evaluated. Importantly, depletion of the cytoplasmic core EJC factor Btz does not result in any observable TE derepression.

Similar results were obtained in cultured ovarian somatic stem cells (OSCs), which are also subject to piRNA-mediated silencing of TEs (Niki et al. 2006; Saito et al. 2009). RNAi-mediated depletion of Mago, Tsu, RnpS1, and Acn, but not of Btz, causes derepression of *gypsy* (Supplemental Fig. S1). Together, our results suggest an involvement of the nuclear EJC plus the factors RnpS1 and Acn in the ovarian piRNA pathway.

### The EJC is required for a post-transcriptional step in piwi expression

**Figure 2.**
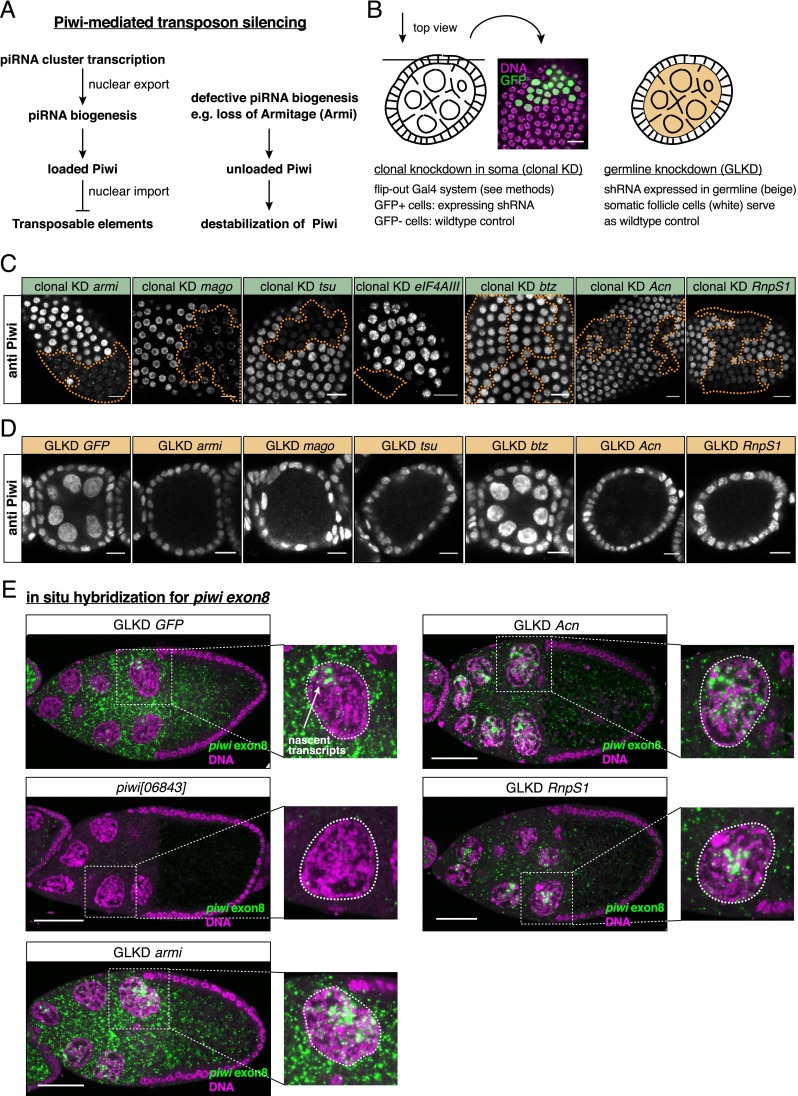
The EJC is required for *piwi* mRNA and protein expression. (*A*) Illustration of Piwi-mediated transposon silencing. piRNA precursor transcripts are expressed from heterochromatic piRNA clusters (e.g., *flamenco*) and exported to cytoplasmic piRNA biogenesis sites. Upon loading with a piRNA, Piwi enters the nucleus, where it guides transcriptional silencing of TEs. Defective piRNA biogenesis (e.g., caused by loss of Armi) prevents Piwi loading, which renders it unstable. (*B*) Illustration of applied RNAi knockdown strategies. Indicated factors were depleted in either clones of somatic cells (*left*) or all germline cells (*right*). Confocal images in *C* and *D* represent surface sections and cross-sections through stage 3–6 egg chambers, respectively. (*C*,*D*) Confocal sections of egg chambers with clones of somatic follicle cells (*C*) or germline cells (*D*) expressing shRNAs against EJC factors stained for Piwi. Bars, 10 μm. Piwi levels are greatly reduced in cells depleted for EJC factors except Btz. shRNAs against *GFP* and *armi* served as controls. (*E*) Confocal sections through stage 9–10 egg chambers expressing shRNAs against the indicated genes in the germline stained for *piwi exon8* (green) and DNA (magenta). Bars, 50 μm. Egg chambers from *piwi[06843]* germline clones served as negative control. Cytoplasmic *piwi* staining is lost upon *Acn* or *RnpS1* knockdown but not upon *armi* knockdown. Nuclear staining (individual nurse cell nuclei enlarged) of *piwi* reflects nascent transcripts and thus active transcription.

To place the EJC into the hierarchy of the piRNA pathway (illustrated in Fig. 2A), we took advantage of the observation that defects in piRNA biogenesis (e.g., loss of Armi) cause severe reductions in Piwi protein levels, presumably because unloaded Piwi is unstable (Fig. 2C; Olivieri et al. 2010; Saito et al. 2010). Interestingly, depletion of Mago, Tsu, RnpS1, or Acn, but not Btz, also causes a strong reduction of Piwi levels in soma and the germline (Fig. 2C,D [see also B for a summary of knockdown strategies]). The clonal knockdown approach in somatic cells allowed us to show that the other nuclear EJC core factor, eIF4AIII, is also required for Piwi accumulation (Fig. 2C).

Because the EJC is known to control RNA fate, we hypothesized that loss of the EJC impairs expression or specification of piRNA precursor transcripts, which are transcribed from discrete, often heterochromatic loci termed piRNA clusters (Senti and Brennecke 2010). However, depletion of the EJC in OSCs results in only moderate and insignificant changes in steady-state RNA levels of *flamenco*, the major somatic piRNA cluster (Brennecke et al. 2007). Also, integrity of the Yb body, the cytoplasmic piRNA processing center in somatic ovarian cells (Szakmary et al. 2009; Olivieri et al. 2010; Saito et al. 2010), does not depend on the EJC (Supplemental Fig. S2).

We therefore analyzed *piwi* expression itself using fluorescent in situ hybridization (FISH). In wild-type egg chambers, an *exon8* antisense probe detected abundant *piwi* transcript in the cytoplasm of nurse cells (Fig. 2E). In addition, signal within nurse cell nuclei was observed in strong foci that presumably reflect nascent transcripts at the sites of transcription. Both signals are absent in *piwi[06843]* mutant egg chambers (germline clones), where a P element inserted into the first intron perturbs *piwi* transcription. Consistent with the loss of Armi resulting in post-translational destabilization of Piwi protein (Olivieri et al. 2010; Saito et al. 2010), *piwi* transcript patterns in the cytoplasm and nucleus are normal in Armi-depleted egg chambers (Fig. 2E). In contrast, depletion of Acn or RnpS1 results in the severe reduction of cytoplasmic *piwi* transcript levels, while nuclear foci are unaffected, indicating that primary *piwi* transcription is normal (cf. the signals in nurse cell nuclei in Fig. 2E). Thus, depleting EJC activity impairs the processing, export, or stability of *piwi* transcripts.

### EJC factors are required for piwi intron4 splicing

**Figure 3.**
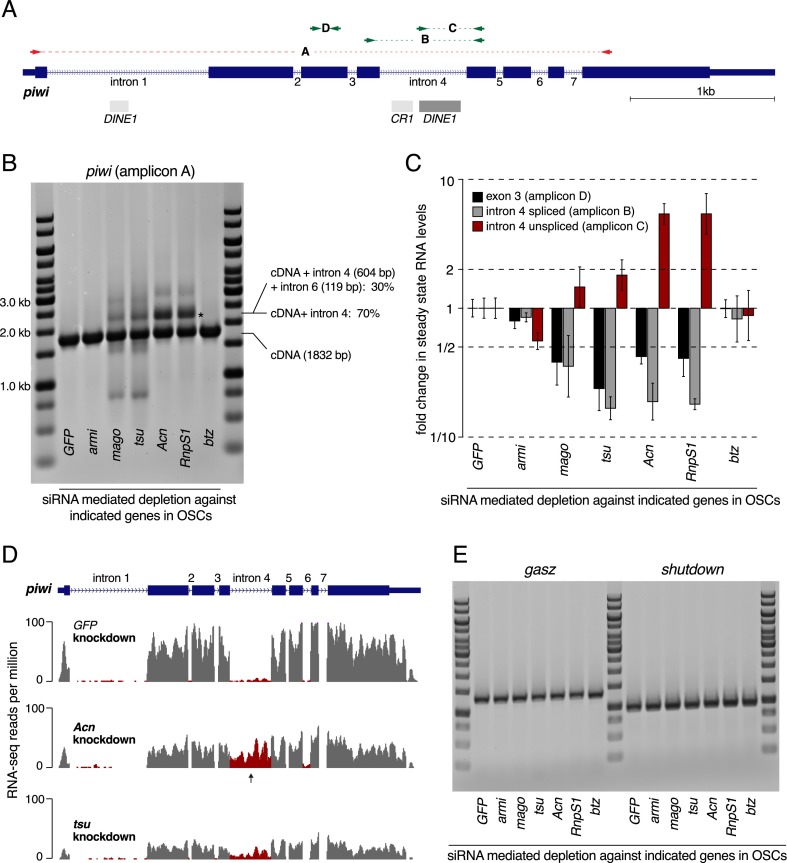
EJC factors are required for *piwi int4* splicing. (*A*) Cartoon of the genomic *piwi* locus showing exons as blue boxes, introns as lines, TE fragments as gray boxes, and the locations of RT–PCR primers used in *B*–*D*. (*B*) Agarose/EtBr gel showing *piwi* RT–PCR products (primers in *exon1* and *exon8*; amplicon A in *A*; size markers at the borders) amplified from total RNA isolated from OSCs depleted for the indicated genes. *piwi* cDNAs containing *int4* or both *int4* and *int6* (indicated by an asterisk) are amplified along with wild-type *piwi* cDNA in *mago*, *tsu*, *Acn*, or *RnpS1* knockdowns. (*C*) Shown are the fold changes in steady-state RNA levels of all *piwi* transcripts (*exon3*; black bars) and those *piwi* transcripts with spliced (gray bars) or unspliced (red bars) *int4* amplified from total RNA isolated from OSCs depleted for the indicated genes. Values are normalized to *rp49* levels, and averages of three biological replicates relative to control knockdowns are shown, with error bars indicating standard deviation. The levels of *piwi* transcripts with spliced and unspliced *int4* are decreased and increased by twofold to fourfold in *mago*, *tsu*, *Acn*, or *RnpS1* knockdowns, respectively. (*D*) RNA-seq profiles at the *piwi* locus obtained from polyA-selected RNA from OSCs depleted for the indicated genes (exonic reads in gray; intronic reads in red; arrows mark *int4*), showing the retention of *int4* in *Acn* and *tsu* knockdowns. (*E*) Agarose/EtBr gel showing *gasz* and *shutdown* RT–PCR products (primers in the respective first and last exons) amplified from total RNA isolated from OSCs depleted for the indicated genes. Unlike *piwi*, the splicing of these transcripts is not affected by the depletion of EJC factors.

Because the nuclear EJC and RnpS1 are required for faithful splicing of numerous mRNAs (Ashton-Beaucage et al. 2010; Roignant and Treisman 2010), we analyzed *piwi* splicing patterns by RT–PCR using total RNA from OSCs depleted for individual EJC factors by RNAi. A primer pair mapping to the first and the last *piwi* exon exclusively amplifies the expected spliced wild-type *piwi* cDNA from control and Armi-depleted cells (Fig. 3A,B, amplicon A).

Depletion of Mago, Tsu, RnpS1, or Acn, but not Btz, results in additional, longer products, suggestive of intron inclusion, and shorter products that are indicative of exon skipping (Mago and Tsu only) (Fig. 3B). We focused on the major longer fragment, considering that it is observed in all four knockdown samples. Sequencing showed that it comprises the expected *piwi* cDNA plus the entire *intron4* (*n* = 10 of 10); three clones contained *intron6* in addition.

Intron retention in cells with reduced EJC activity was unexpected because loss of the EJC has previously been shown to be associated with skipping of exons, in particular those adjacent to very long introns (Ashton-Beaucage et al. 2010; Roignant and Treisman 2010). We therefore confirmed the retention of *piwi intron4* (*int4*) by qPCR. Compared with control cells, we observed an approximately fourfold decrease of the spliced species (amplicon B) and an approximately fourfold increase of the unspliced species (amplicon C) in Acn- or RnpS1-depleted cells (Fig. 3C). The accumulation of the unspliced species is evident but less pronounced in Mago- or Tsu*-*depleted cells, perhaps because depletion of these core factors has a broader impact on *piwi* splicing patterns or causes a general reduction in cell viability. We observed a similar degree of *int4* retention also in ovaries depleted for EJC factors specifically in germline cells (Supplemental Fig. S3).

A highly similar intron retention pattern was found by sequencing polyA-selected RNAs: *int4* is substantially retained, and *int6* is slightly overrepresented in Acn- or Tsu-depleted OSCs (Fig. 3D). These data also confirm a general reduction in *piwi* steady-state RNA levels, especially in Tsu-depleted cells, presumably a consequence of nonsense-mediated decay (NMD) because inclusion of *int4* adds a premature stop codon.

Intron retention is not a general phenotype in EJC-depleted cells; splicing of several other transcripts (e.g., of the piRNA pathway factors *gasz* or *shutdown*) is normal in the various OSC knockdowns tested (Fig. 3E). Thus, the reduced Piwi activity is probably due to a selective failure of *piwi* RNA splicing.

### piRNA pathway defects in EJC-depleted ovaries are largely due to retention of piwi intron4

To check whether *int4* retention is indeed the major block to Piwi expression in EJC-depleted cells, we tested whether removing the intron renders *piwi* EJC-independent. We generated transgenic flies that express an N-terminal GFP-Piwi fusion in the context of a fully functional genomic construct containing the entire *piwi* locus (*GFP-piwi*) (Sienski et al. 2012) and flies expressing an equivalent gene that lacks *int4* (*GFP-piwi[Δint4]*).

**Figure 4.**
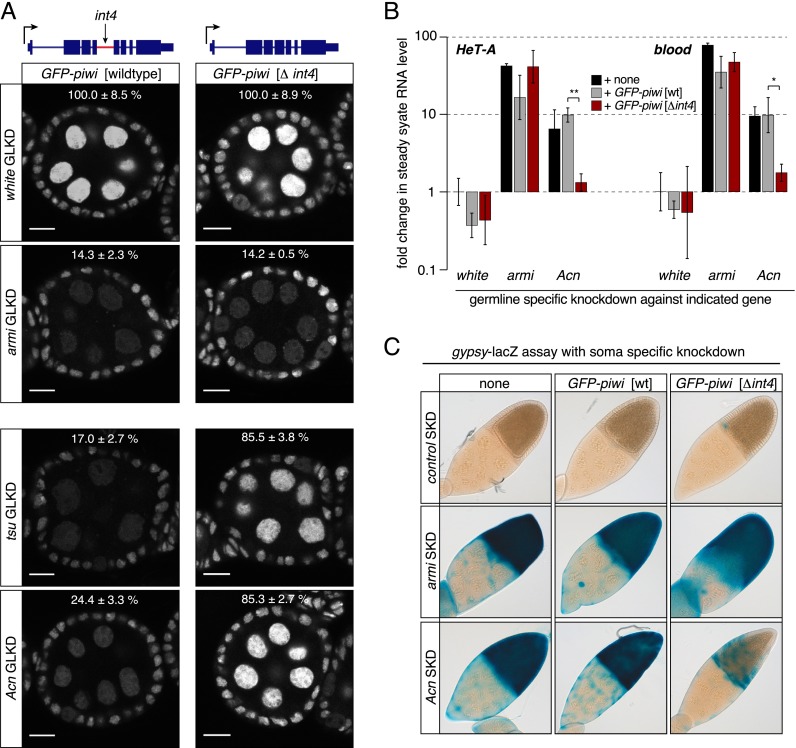
A *piwi* transgene lacking *int4* rescues transposon derepression in EJC-depleted cells. (*A*) Expression of wild-type *GFP-piwi*, but not of *GFP-piwi* Δ*int4*, is dependent on EJC factors. Shown are confocal sections through egg chambers expressing *GFP-piwi* or *GFP-piwi* Δ*int4* depleted for the indicated genes in germline cells. Bars, 10 μm. Relative GFP levels (±standard deviation) from the indicated *GFP-piwi* transgenes in comparison with the *white* control knockdown are indicated (GFP fluorescence levels in nurse cell nuclei normalized to those in follicle cell nuclei from three egg chambers). (*B*) Displayed are fold changes in steady-state RNA levels of *HeT-A* and *blood* in ovaries depleted for the indicated genes in the germline and expressing the indicated *GFP-piwi* transgenes. RNA levels are normalized to *rp49* levels, and averages of three biological replicates relative to control knockdowns are shown. Error bars indicate standard deviation. (*) *P* < 0.01; (**) *P* < 0.001. TE derepression caused by *Acn* knockdown is rescued by *GFP-piwi* Δ*int4* but not by wild-type *GFP-piwi*. (*C*) Shown are the X-gal stainings of egg chambers depleted for the indicated genes in somatic follicle cells and expressing the *gypsy*-*lacZ* reporter and the indicated *GFP-piwi* transgenes. *GFP-piwi* lacking *intron4*, but not wild-type *GFP-piwi*, greatly reduces the *gypsy-lacZ* expression induced by the depletion of *Acn*.

As expected, GFP-Piwi levels from the former construct are sensitive to the depletion of EJC factors in ovarian somatic and germline cells (Fig. 4A; Supplemental Fig. S4A). In contrast, GFP-Piwi from *GFP-piwi[Δint4]* is insensitive to depletion of Tsu or Acn (Figs. 4A; Supplemental Fig. S4A) while remaining sensitive to reduced piRNA synthesis by Armi depletion. Retention of *int4* is therefore causal to the reduced Piwi levels in EJC-depleted cells.

We further tested whether the EJC-independent *GFP-piwi[Δint4]* transgene rescues the TE derepression phenotype in EJC-depleted flies. We depleted Acn specifically in ovarian germline or somatic cells of flies carrying either the wild-type *GFP-piwi* or the *GFP-piwi[Δint4]* construct. TE derepression in the germline caused by the depletion of Acn, but not of Armi, is greatly rescued by the *GFP-piwi[Δint4]* transgene and not by the wild-type *GFP-piwi* transgene (Fig. 4B). The transgene also rescues TE derepression in the soma: In Acn-depleted somatic cells, expression of the *gypsy-lacZ* reporter is largely repressed in the presence of *GFP-piwi[Δint4]* (Fig. 4C).

The *GFP-piwi[Δint4]* construct fails to rescue germline TE derepression due to Tsu depletion (data not shown), perhaps because these flies also show reduced germline expression of AGO3 (Supplemental Fig. S4B), a second central Argonaute protein involved in the piRNA pathway. *AGO3* pre-mRNA has very large introns and thus resembles transcripts known to require the EJC for efficient splicing. Indeed, *AGO3* mRNA level is reduced upon Tsu but not Acn depletion. We did not detect misspliced *AGO3* mRNA species, possibly due to efficient NMD (Supplemental Fig. S4C,D). We note that the *GFP-piwi[Δint4]* construct does reduce desilencing of *mdg1* in Tsu-depleted follicle cells in which AGO3 is not expressed (Supplemental Fig. S4E). Taken together, our results indicate that missplicing of *piwi int4* is the major cause of TE derepression in ovaries depleted for EJC components.

### Splicing of piwi intron4 requires deposition of the EJC at a nearby splice junction

**Figure 5.**
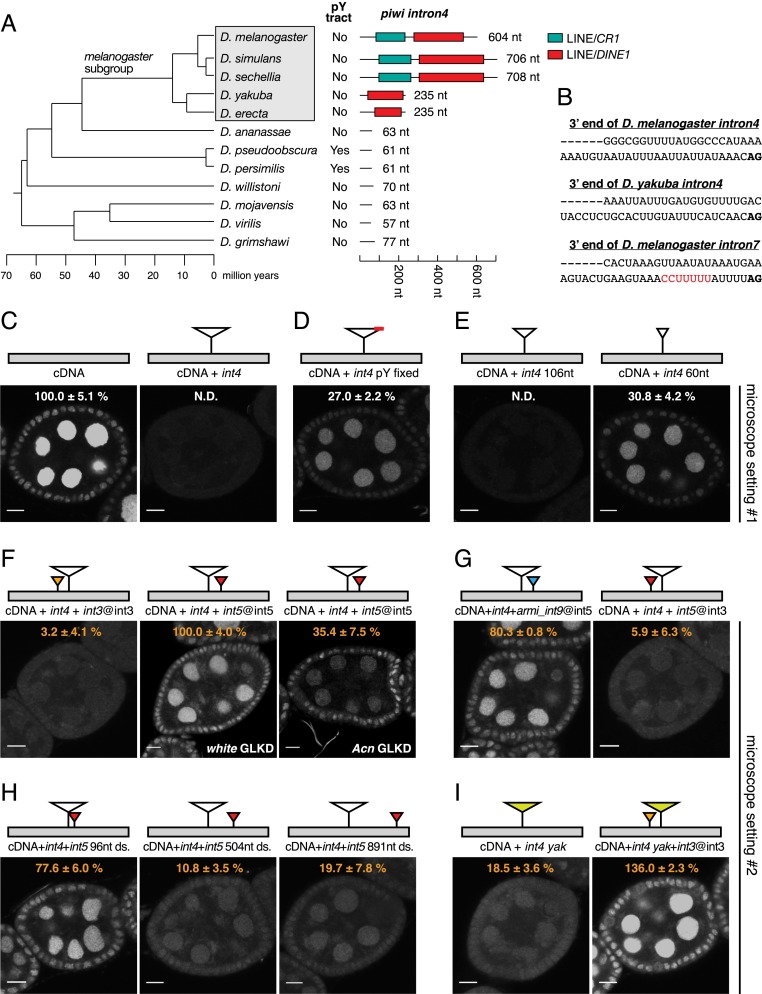
The EJC facilitates *piwi int4* splicing from a nearby splice junction. (*A*) Architecture of *piwi int4* from 12 *Drosophila* species (phylogenetic tree is at the *left*). Indicated are TE fragments (colored), intron length, and the presence of a pY tract. *piwi int4* is enlarged in the *melanogaster* subgroup (boxed) due to insertions of TE fragments. (*B*) Depicted are the last 50 nt of the indicated introns, with pY tract in red. *piwi int4* from *D. melanogaster* and *D. yakuba* have suboptimal pY tracts. (*C–I*) *piwi int4* is not efficiently spliced when it is placed in isolation. The features of *int4* that contribute to the inefficient splicing and the effects of additional flanking introns are examined. Shown are confocal sections through egg chambers expressing the indicated *GFP-piwi* transgenes (depicted as cartoons *above* each image). Bars, 10 μm. In *F*, shRNAs against *white* or *Acn* are expressed in germline cells (GLKD). The transgene cartoons represent the *piwi* cDNA (gray), *melanogaster int4* (white large triangles), *yakuba int4* (green large triangles), and additional indicated introns (small colored triangles). The indicated GFP levels (±standard deviation) were calculated by measuring GFP fluorescence in nurse cell nuclei of three egg chambers. Microscope settings were identical in *C*–*E* and *F*–*I*, respectively. GFP intensity was set to 100% for the *left* panel in *C* and the *middle* panel in *F*. For a global comparison, the intensity of *GFP-piwi* [cDNA + *int4* + *int5*@int5] is 13.0 ± 2.0% of the intensity of *GFP-piwi* [cDNA].

To understand why splicing of *piwi int4* is sensitive to EJC levels, we compared the *int4* sequence in *Drosophila melanogaster* with that in other *Drosophilids* (Fig. 5A). In the *melanogaster* subgroup, *int4* harbors one or two TE remnants that extend the intron to ∼200–700 nt, whereas the intron is devoid of TE fragments and very short (∼60 nt) in the more distantly related species. Also, the 3′ end of *piwi int4* lacks a robust polypyrimidine tract (pY; defined as at least seven consecutive pyrimidines) in nearly all of these species. Assembly of spliceosome components at 3′ splice junctions involves recognition of the pY tract by the U2 snRNP factor U2AF65 (Zamore et al. 1992). Introns with a poor pY tract can be spliced in vitro, but only if they are <90 nt (Guo et al. 1993).

This analysis raised the possibility that *int4* is a suboptimal intron in the *melanogaster* subgroup. To test this idea, we generated transgenic flies harboring the aforementioned genomic *GFP*-*piwi* construct but lacking either all introns or all except *int4*. The intron-less construct drives readily detectable GFP-Piwi expression in ovarian somatic and germline cells (Fig. 5C). However, addition of *int4* abolishes expression of GFP-Piwi almost completely (Fig. 5C), indicating that even wild-type cells cannot splice *piwi int4* if it is present in isolation.

Replacing the 3′-most 50 nt of *int4* with the equivalent part of *piwi int7* that contains a clear pY tract (the *pY-fixed* construct) (Fig. 5B) largely rescues *int4* splicing, as does shortening of *int4* to 60 nt (Fig. 5D,E). A length reduction to 106 nt does not restore splicing, consistent with the aforementioned length cutoff of ∼90 nt for introns with poor pY tracts (Guo et al. 1993). These results suggest that *piwi int4* is a very poorly defined intron due to the lack of an optimal pY tract coupled with its large size.

Because *piwi int4* is efficiently spliced in the context of the wild-type locus but poorly spliced when isolated, perhaps prior splicing of flanking introns is required for *int4* definition. We tested this idea directly by adding either *int3* or *int5* at their natural positions to the transgene that contains only *int4*. Addition of *int3* has no measurable impact, but the presence of *int5* results in significant GFP-Piwi expression to about half the level observed in the *pY-fixed* construct (Fig. 5F). Most importantly and as shown for the wild-type construct, GFP-Piwi expression from the *int4/5* construct is EJC-dependent, being approximately threefold reduced in germline cells upon germline-specific depletion of Acn (Fig. 5F).

These results strongly favor a model in which splicing of *int5* with subsequent EJC deposition facilitates *int4* splicing. To understand why addition of *int5* but not of *int3* rescues *int4* splicing, we analyzed the impact of intron identity, absolute intron distance to *int4*, and relative intron positioning (upstream versus downstream) on GFP-Piwi expression. Intron identity plays no role because an intron derived from the *armi* locus rescues expression as efficiently as *piwi int5*, while *piwi int5* placed at the *int3* position lacks rescuing activity (Fig. 5G). Increasing the distance between *int4* and *int5* from 200 nt (wild type) to 500 or 900 nt impairs rescue, while shortening the distance has no impact (Fig. 5H), suggesting that proximity to the splice junction is important. To test whether the upstream introns fail to rescue because they are too distant from the *int4* 3′end, we examined whether an upstream intron can rescue the splicing of a shorter *int4* from *Drosophila yakuba* (235 nt), which, when isolated, is also splice-defective in wild-type cells (Fig. 5I). This shorter *int4* is indeed rescued by the upstream presence of the *melanogaster int3* (Fig. 5, cf. I and F), indicating that both upstream and downstream introns can rescue *int4* splicing if placed nearby, presumably due to prior deposition of the EJC to a flanking exon–exon junction.

### Splicing of first introns tends to depend on the EJC if followed by a large second intron

To investigate whether *piwi int4* is indicative of a more general role of the EJC in efficient intron removal, we sequenced polyA-selected RNAs from OSCs depleted for Tsu, Acn, or GFP (Materials and Methods). We considered ∼1500 introns for which a significant quantitative analysis was possible (Materials and Methods).

**Figure 6.**
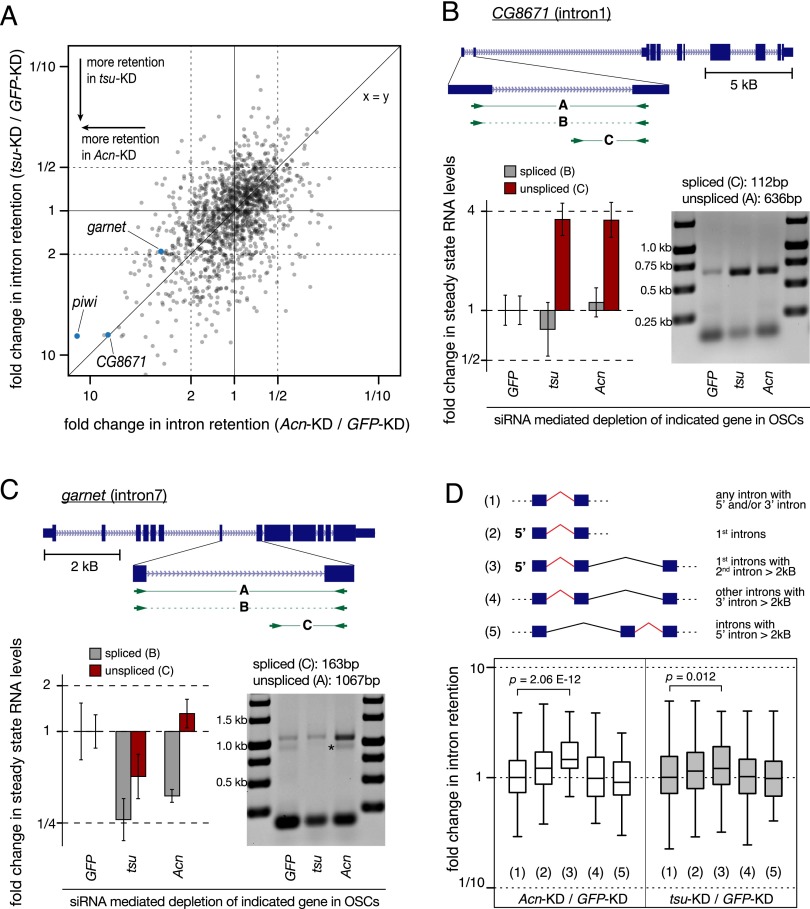
Transcriptome-wide analysis of EJC-dependent introns. (*A*) Scatter plot showing the fold changes in the retention of individual introns of OSC transcripts upon *Acn* or *tsu* knockdown relative to the control knockdown. Validated hits are indicated (blue dots). *piwi int4* is among the most prominent introns that are retained both in *Acn* and *tsu* knockdowns. (*B*,*C*) Experimental validations of enhanced retention of *CG8671 int1* and *garnet int7* in *tsu*- or *Acn*-depleted OSCs using polyA-selected RNAs. Shown at the *top* is a cartoon of the respective locus (exons as boxes and introns as lines) with the location of the primers in RT–PCR. The bar charts indicate fold changes in steady-state levels of the indicated *CG8671* (*B*) or *garnet* (*C*) transcripts. (Gray) Spliced; (red) unspliced. Amplicon identity is given *above*. The Agarose/EtBr gel images show RT–PCR products obtained with primers mapping to the flanking exons. The asterisk in *C* marks a nonspecific product. (*D*, *top*) The cartoon depicts the analyzed groups of introns, considering their location in the transcript and the identity of flanking introns. The box plot shows fold changes in intron retention for the groups defined *above* upon *Acn* or *tsu* depletion. First introns followed by a large second intron (group 3) are significantly more retained in EJC-depleted cells (*P*-values are calculated by the Wilcoxon rank-sum test).

An analysis of intronic RNA sequencing (RNA-seq) reads indicates that splicing of most introns is EJC-independent (median changes in intron retention index upon *tsu* and *Acn* knockdown are both 1.00). Nevertheless, 286 introns exhibit pronounced retention characteristics (twofold to 10-fold higher than in control cells) in cells depleted for either Tsu or Acn (Fig. 6A). We verified the RNA-seq results for several EJC-dependent introns by performing RT experiments or by qPCR using amplicons that discriminate spliced and nonspliced transcripts (shown for *CG8671* and *garnet* in Fig. 6B,C). Of note, *piwi int4* is among the most affected intron transcriptome-wide.

As a poor pY tract is an apparent characteristic of *piwi int4*, we inspected whether EJC-dependent introns generally exhibit a weak pY tract. However, the group of introns harboring a poor pY tract (*n* = 272) (Materials and Methods) is not significantly more dependent on the EJC than the average intron (median changes in intron retention index upon *tsu* or *Acn* knockdown are 1.03 and 1.02, respectively; *P*-values > 0.5). Also, the suspicious TE insertions in *piwi int4* do not seem to suggest a general pattern: TE remnants are only rarely found in *tsu*- or *Acn*-dependent introns (only four out of the 30 most affected introns harbor TE remnants).

Loss of the nuclear EJC has been shown to result in exon skipping for transcripts containing large introns (Ashton-Beaucage et al. 2010; Roignant and Treisman 2010). This appears not to be the case for intron retention because the intron retention index is not significantly increased for either introns that are themselves >2 kb (*n* = 162; median changes in intron retention index upon *tsu* or *Acn* knockdown are 0.93 and 0.92, respectively) or introns within transcripts containing at least one intron >10 kb (*n* = 353; median changes in intron retention index upon *tsu* or *Acn* knockdown are 1.00 and 0.94, respectively).

Manual inspection of many gene loci containing an EJC-dependent intron indicated that affected introns are often first introns and that they are frequently followed by a large second intron (e.g., *CG8671* in Fig. 6B; see also Supplemental Table S1). The population of first introns followed by a large second intron (>2 kb; *n* = 77) is significantly more likely to be inefficiently spliced in *tsu*-depleted (median = 1.21; *P* = 0.012) or *Acn*-depleted (median = 1.46; *P* = 2.06 × 10^−12^) cells compared with control cells (Fig. 6D). In comparison, the group of all first introns (*n* = 320) or internal introns immediately followed (*n* = 64) or preceded (*n* = 99) by a large intron shows no significantly different behavior in EJC-depleted cells compared with control cells (Fig. 6D). Together, our results demonstrate an unanticipated dependency of first introns that are followed by a large second intron on the EJC for their splicing.

### Exon skipping and intron retention are genetically separable events

Both exon skipping and intron retention are defects in EJC-depleted cells, arguing that the two processes are mechanistically related. However, we noticed one clear difference between the two phenotypes: Loss of Acn strongly impacts intron retention yet was not previously reported to have an impact on exon skipping (Ashton-Beaucage et al. 2010; Roignant and Treisman 2010).

**Figure 7.**
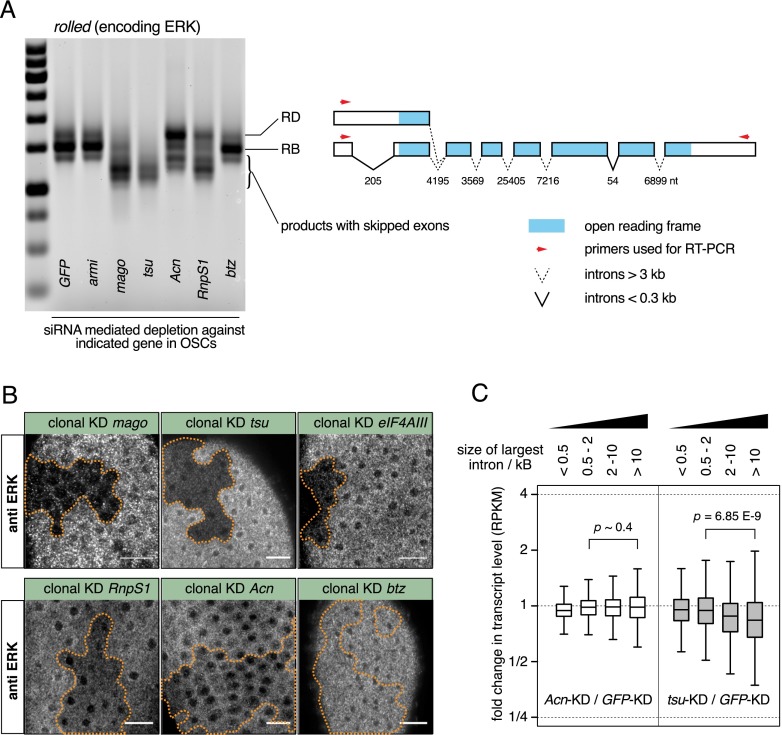
Depletion of core EJC factors but not *Acn* results in exon skipping. (*A*) Agarose/EtBr gel image showing RT–PCR products of *rolled* transcripts amplified from total RNA from OSCs depleted for the indicated genes. Illustrated on the *right* is the *rolled* locus showing exons as boxes, with the ORF depicted in blue, introns as solid (<0.3 kb) and dashed (>3 kb) lines, and arrows as primers used in RT–PCR (modified from Ashton-Beaucage et al. 2010, © 2010, with permission from Elsevier). Each intron is labeled with their size (in nucleotides). *rolled*-RB is the major product in wild-type cells, and *rolled*-RD is an annotated *rolled* transcript with *intron1* retained that still contains the wild-type ORF. Knockdown of *mago*, *tsu*, and, to a lesser degree, *RnpS1* causes exon skipping of *rolled*, whereas the splicing is largely unaffected by the knockdown of *Acn*. (*B*) Confocal sections through the follicular epithelium of stage 3–6 egg chambers stained for ERK (encoded by *rolled*), showing that the depletion of *mago*, *tsu*, or *eIF4AIII* leads to a severe reduction in ERK protein level, which is moderately or only very mildly affected by the depletion of *RnpS1* or *Acn*, respectively. Bars, 10 μm. Cells within the clone (boundaries marked by dashed line) express shRNAs against the indicated genes. (*C*) Box plot showing fold changes in the steady-state RNA level of transcript groups containing increasing intron length upon *Acn* (*left*) or *tsu* (*right*) depletion in OSCs. Grouping of transcripts was according to the largest intron (*x* in kilobases): from *left*, 0 < *x* = <0.5; 0.5 < *x* = 2; 2 < *x* = < 10; 10.0 < *x*. The level of transcripts with large introns is decreased upon depletion of *tsu* but not of *Acn* (*P*-values based on Wilcoxon rank-sum test).

We therefore analyzed splicing patterns of the large intron-containing *rolled* and *light* transcripts in OSCs depleted for EJC core factors or RnpS1 or Acn. Consistent with previous reports, depletion of Mago or Tsu leads to severe exon skipping (Fig. 7A; Supplemental Fig. S5; Ashton-Beaucage et al. 2010; Roignant and Treisman 2010). The same is true, although to a lesser extent, for depletion of RnpS1 but not for Acn, whose depletion results in very mild exon skipping (Fig. 7A; Supplemental Fig. S5). Similarly, protein levels of the Rolled MAP kinase ERK are strongly reduced in vivo in cell clones depleted for EJC core factors (Mago, Tsu, and eIF4AIII), only mildly reduced upon RnpS1 depletion, and almost wild type in Acn-depleted cells (Fig. 7B). In contrast, loss of any involved EJC factor affects Piwi protein levels equally under comparable experimental conditions (Fig. 2C,D).

As exon skipping results in reduced levels of affected transcripts (presumably due to NMD), depletion of the core EJC affects preferentially levels of transcripts harboring long introns (Ashton-Beaucage et al. 2010; Roignant and Treisman 2010). We confirmed that transcript levels are increasingly more affected as a function of increasing intron length upon Tsu depletion in OSCs (Fig. 7C). Again, no such trend is detectable upon Acn depletion, confirming its limited impact on exon skipping.

Taken together, our observations indicate that exon skipping and intron retention are related yet genetically separable processes. While depletion of nuclear core EJC factors results in both phenotypes, depletion of Acn only has a clear effect on the intron retention phenotype.

## Discussion

Genetic screens have uncovered a surprising link between the EJC and the piRNA pathway in *Drosophila*. We dissected the underlying molecular cause and uncovered several principles that connect the EJC to intron definition and splicing. The major outcome of our study is as follows:

(1) Loss of the nuclear EJC or the accessory factors RnpS1 or Acn impairs the *D. melanogaster* piRNA pathway due to impaired splicing of the *piwi* transcript.
(2) Deposition of the EJC to flanking introns, but not direct recruitment by the spliceosome, underlies the EJC’s impact on splicing.
(3) Many introns—preferentially first introns followed by large second introns—require the EJC for their splicing.
(4) Exon skipping and intron retention depend on related but distinct aspects of EJC function.

### Roles of the EJC in the piRNA pathway

Defective splicing of *piwi int4* is a major molecular defect underlying the involvement of the EJC in the *D. melanogaster* piRNA pathway (Figs. 3, 4). During evolution, one or two LINE fragments appear to have inserted into *int4*, which, together with its poor pY tract, renders it too long to be spliced efficiently. Based on these criteria, an involvement of the EJC in *piwi* expression appears restricted to the *melanogaster* subgroup (which branched from the *ananassae* subgroup ∼45 million years ago) (Fig. 5; Tamura et al. 2004). It is unclear whether a suboptimal *int4* is simply tolerated given its rescue via the EJC or whether the dependence of *int4* on the EJC provides a selective advantage.

In the case of Acn depletion, TE repression is nearly entirely restored by expression of an EJC-independent *piwi* transgene. Loss of Tsu or Mago, on the other hand, results in additional defects that impair TE silencing. It is currently unclear whether splicing of other piRNA pathway factor transcripts requires the core EJC. A strong candidate is AGO3, which is expressed as an ∼150-kb pre-mRNA with several large introns; indeed, AGO3 transcript levels are affected upon depletion of core EJC but not peripheral EJC factors (Supplemental Fig. S4).

### EJC assembly to flanking splice junctions occurs prior to its contribution to splicing

Because deposition of the minimal EJC core (pre-EJC: eIF4AIII, Mago, and Tsu) at the upstream exon occurs shortly before or during exon ligation and therefore depends on a successful splicing event (Gehring et al. 2009), most studies on the EJC have focused on its role in controlling mRNA fate downstream from splicing. It came as a surprise that the EJC also impacts the process of splicing itself (Ashton-Beaucage et al. 2010; Roignant and Treisman 2010). However, analyzing the molecular basis for this contribution to splicing has been difficult due to the complex architecture of previously described target transcription units (long genes with large introns harboring numerous TE insertions, often situated within the heterochromatin). In particular, it was unclear whether the role of the EJC in the splicing of transcripts containing large introns reflects a noncanonical role of EJC components in splicing or whether canonically deposited EJCs at flanking splice junctions aid the definition of flanking introns (discussed in Ashton-Beaucage and Therrien 2011).

The finding that the EJC is also required for splicing of *int4* at the compact and euchromatic *piwi* locus provides a powerful system to address this important question. Our collective data strongly indicate that conventional assembly of the EJC at splice junctions can facilitate the subsequent splicing of neighboring introns that pose a challenge for the spliceosome, such as ones that are atypically large (Ashton-Beaucage et al. 2010; Roignant and Treisman 2010) or lack strong splicing signals (this study). In the case of *piwi int4*, a neighboring EJC (upstream or downstream) can assist the splicing if it is bound within ∼250 nt of the 3′ splice site (Fig. 5F,H). Interestingly, this distance requirement resembles that of the minimum size of an intron in the intron definition model of splicing (Fox-Walsh et al. 2005) and indicates a direct role of the EJC in aiding splicing rather than a licensing model in which EJC deposition anywhere on the message promotes splicing of suboptimal introns. The EJC has been shown to interact with a number of SR proteins (Singh et al. 2012), suggesting that the EJC acts as an RNA-bound scaffold to recruit splicing machineries via SR proteins.

### A temporal order in splicing instructed by the EJC?

Previous observations point to a preferential role of the EJC in the splicing of complex transcripts; namely those that contain unusually large introns, often harboring TE insertions. Why, however, would first introns followed by large second introns be disproportionally dependent on the EJC (Fig. 6D)? These first introns themselves are not unusually large and exceptional, yet their dependency on a flanking EJC suggests that they are intrinsically difficult to splice. An attractive, albeit speculative, model is that evolution has selected this dependency in order to splice first introns only after successful splicing of the large downstream intron. Large introns are likely to contain cryptic polyadenylation/cleavage sites, and their usage is suppressed by deposition of U1 onto the nascent transcript (Kaida et al. 2010). Delaying intron splicing to a time point after transcription and splicing of the large downstream intron would therefore increase gene expression fidelity. Interestingly, first introns have been reported to be less efficiently spliced compared with internal introns in *Drosophila* (Khodor et al. 2011).

A related hypothesis is that the EJC licenses splice events during alternative splicing. Alternatively spliced introns often have weak splice recognition sites (Clark and Thanaraj 2002; Garg and Green 2007), and it has been shown that splicing of neighboring introns influences the selection of alternative splice sites (Fededa et al. 2005; Glauser et al. 2011). It would be intriguing if the order of intron splicing impacts alternative splicing in an EJC-dependent manner, thereby increasing the transcript repertoire in higher organisms.

### Exon skipping versus intron retention and the role of Acinus

Intron retention and exon skipping have two different molecular causes: defective intron and exon definition, respectively (De Conti et al. 2013). Nevertheless, depletion of core EJC factors (eIF4AIII, Mago, and Tsu) or of RnpS1 can give rise to both phenotypes, suggesting that they are linked (Ashton-Beaucage et al. 2010; Roignant and Treisman 2010; this study). Indeed, we hypothesize that both phenotypes manifest after a first successful round of splicing. In the case of exon skipping, we propose that the reinforcement of the exon definition of the newly formed exon pair is compromised (see also Ashton-Beaucage and Therrien 2011). In the case of intron retention, we propose that EJC loss results in a failure to license a weak nearby splice site.

Despite these similarities, it appears that these two processes are at least to some extent distinct, as loss of Acn leads more or less exclusively to intron retention and only marginally to exon skipping (Fig. 7). Our genome-wide analysis shows a global correlation between intron retention levels in Acn- and Tsu-depleted cells (Fig. 6A). Also, Acn enhances the splicing of *piwi int4* in an EJC-dependent manner (Fig. 5F). Assembly of the EJC core is therefore probably a prerequisite for Acn to help defining neighboring splice sites. Acn forms a stable complex with the SR protein RnpS1 to interact with the EJC (Tange et al. 2005) and has recently been implicated in the regulation of alternative splicing (Michelle et al. 2012). Further genetic and biochemical analyses on Acn (and RnpS1) should be the key to understanding their distinctive roles in intron definition and exon definition.

## Materials and methods

### Fly husbandry and strains

All flies were kept at 25°C. *MTD-GAL4* and *piwi[06843]* strains were obtained from the Bloomington *Drosophila* Stock Center, *tj*-*GAL4* strain was obtained from the *Drosophila* Genomics Resource Center. The *lacZ* sensor flies for *Burdock* and *gypsy* were described in Handler et al. (2013) and Sarot et al. (2004), respectively. shRNA lines were generated by cloning short hairpins (for sequences, see Supplemental Table S3) into the *Valium-20* vector modified with a *white* selection marker (Ni et al. 2011) and inserting these into the *attp2* landing site on chromosome 3 (Groth et al. 2004). *GFP-Piwi* flies were described in Sienski et al. (2012). Knockdown crosses with Vienna *Drosophila* Resource Center lines (Dietzl et al. 2007) were with a *UAS-Dcr2; NGT Gal4; nosGal4* driver line (Handler et al. 2013). shRNA lines were used for the *Act-Gal4* flip-out clones (Olivieri et al. 2010) except for *fs(1)Yb*, for which a Vienna *Drosophila* Resource Center line (GD25437) was used.

For all experiments, flies were aged for 4–7 d. Knockdown experiments were always accompanied by a negative control of the same genetic background (carrying an shRNA against either *GFP* or *white*). During the last 2 d, flies were kept on apple juice plates supplemented with yeast paste at medium density to promote egg laying and uniform ovary morphology.

### Cell culture

OSCs were cultured as described (Niki et al. 2006; Saito et al. 2009). In each experiment, 3 × 10^6^ to approximately 4 × 10^6^ cells were transfected twice with 200 pmol of preannealed siRNAs using Cell Line Nucleofector kit V (Amaxa Biosystems, program T-029) and cultured for 4–5 d (2 d plus 2–3 d) before harvesting (see Supplemental Table S4 for siRNA sequences).

### Pacman[GFP-piwi] constructs

The construction of *GFP-piwi* was described previously (Sienski et al. 2012). All other GFP*-piwi Pacman* plasmids were constructed via Gibson assembly (Gibson et al. 2009). *D. yakuba* genomic DNA was obtained from the *Drosophila* Species Stock Center. The 3′ end of *D. melanogaster piwi intron4* was replaced with the sequence from *piwi intron7* using gBlock DNA fragments (Integrated DNA Technologies). The resulting sequences of shortened intron4 are as follows: *intron4*_60nt, **GT**AAGACTTTAAACTATATTTAAAT-deletion-CCATAAAAAATGTAATATTTAATTATTATAAAC**AG**; and *intron4*_106nt, **GT**AAGACTTTAAACTATATTTAAATTAACAAGCTCTTGTGTCGCAAAC-deletion-GCAGTTTAGGGCGGTTTTATGGCCCATAAAAAATGTAATATTTAATTATTATAAAC**AG**.

### Immunohistochemistry, RNA FISH, and image analysis

Rabbit α-Ago3 was described in Brennecke et al. (2007). Mouse monoclonal α-Piwi was raised against the first 150 N-terminal amino acids.

In situ hybridization was performed as described in Lecuyer et al. (2007). Digoxigenin (DIG)-labeled probes were in vitro transcribed from *piwi exon8* cloned into pBluescriptII using the following primers: 5′-ACTGACaagcttTGCCTCAATGGATCTACAGCAAA-3′ and 5′- ACTGACggtaccCGAACTTGTTGCGAGACCAGATA-3′. In situ signal was developed using fluorescein tyramide conjugate (Perkin Elmer).

All fluorescent images were taken on an LSM 780 (Zeiss). GFP intensity was quantified by ImageJ version 1.48f (Figs. 4, 5; Supplemental Fig. S3).

### RT–PCR analysis

Total RNA was isolated from OSCs or ovaries using Trizol (Ambion). cDNA was prepared via random priming of total RNA unless otherwise stated. qPCR was performed using standard techniques. All experiments were performed in biological triplicate with technical duplicates. Relative RNA levels were normalized to *rp49* levels. Fold enrichments were calculated in comparison with respective RNA levels obtained from flies expressing a control hairpin or from control siRNA transfections into OSCs (Schmittgen and Livak 2008). Primer sequences for qRT–PCR and RT–PCR analyses are listed in Supplemental Tables S5 and S6.

### RNA-seq

PolyA-plus or rRNA-depleted RNA from biological duplicates of OSCs treated with siRNA against *GFP*, *tsu*, or *Acn* was selected with Dynabeads Oligo(dT) (Invitrogen) from total RNA, fragmented, and reverse-transcribed with random hexamers. Strand-specific libraries were prepared using the UDG digestion-based strategy (Zhang et al. 2012) and cloned with NEBNext ChIP-seq library preparation reagent set for Illumina (New England Biolabs), and 50-bp paired-end reads were sequenced on a HiSeq2000 (Illumina) instrument. This yielded 10 million–20 million genome-mappable reads per sample. For the computational analyses, we first extracted high-quality bases from every read (6–50 nt) and mapped these to the *Drosophila* genome as well as to the transcript and intron annotations in FlyBase release 5.56, allowing up to three mismatches. Uniquely aligned reads were used for quantification of gene expression levels and intron levels. Transcripts that scored >10 RPKM (reads per kilobase per million) in *GFP* knockdown (*n* = 4203) and introns whose RPKM values were >10% of their transcript RPKM value in any of biological duplicates (*n* = 1478) were analyzed. The intron retention index was calculated as the intron RPKM level divided by the transcript RPKM level in the respective samples.

### Statistical analysis

We used statistical packages implemented in R 2.15.3 for all calculations and plots. For data visualization in box plot format, we used standard features (horizontal bars represent median, the box depicts 25th and 75th percentile, and whiskers represent the 1.5 interquartile range). Statistical significances in Figures 6D and 7D were computed with Mann-Whitney *U*-test; other *P*-values were calculated using Student’s *t*-test.
